# Implementing patient-reported outcomes in routine clinical care for diverse and underrepresented patients in the United States

**DOI:** 10.1186/s41687-022-00428-z

**Published:** 2022-03-07

**Authors:** Colby J. Hyland, Ruby Guo, Ravi Dhawan, Manraj N. Kaur, Paul A. Bain, Maria O. Edelen, Andrea L. Pusic

**Affiliations:** 1 Harvard Medical School, Brigham and Women’s Hospital, Boston, MA USA; 2 Harvard School of Public Health, Brigham and Women’s Hospital, Boston, MA USA; 3 Harvard Medical School, Countway Library, Boston, MA USA

**Keywords:** Patient-reported outcome, Patient-reported outcome measure, PRO, PROM, Clinical care, Implementation, Diverse, Underrepresented patient population

## Abstract

**Background:**

Patient-reported outcomes (PROs) are used increasingly in routine clinical care and inform policies, reimbursements, and quality improvement. Less is known regarding PRO implementation in routine clinical care for diverse and underrepresented patient populations.

**Objective:**

This review aims to identify studies of PRO implementation in diverse and underrepresented patient populations, elucidate representation of clinical specialties, assess implementation outcomes, and synthesize patient needs, concerns, and preferences.

**Methods:**

MEDLINE, Embase, Web of Science, CINAHL, and PsycINFO were searched September 2021 for studies aiming to study PRO implementation in diverse and underrepresented patient populations within the United States. Studies were screened and data extracted by three independent reviewers. Implementation outcomes were assessed according to Proctor et al. taxonomy. A descriptive analysis of data was conducted.

**Results:**

The search yielded 8,687 records, and 28 studies met inclusion criteria. The majority were observational cohort studies (n = 21, 75%) and conducted in primary care (n = 10, 36%). Most studies included majority female (n = 19, 68%) and non-White populations (n = 15, 54%), while fewer reported socioeconomic (n = 11, 39%) or insurance status (n = 9, 32.1%). Most studies assessed implementation outcomes of feasibility (n = 27, 96%) and acceptability (n = 19, 68%); costs (n = 3, 11%), penetration (n = 1, 4%), and sustainability (n = 1, 4%) were infrequently assessed.

**Conclusion:**

PRO implementation in routine clinical care for diverse and underrepresented patient populations is generally feasible and acceptable. Research is lacking in key clinical specialties. Further work is needed to understand how health disparities drive PRO implementation outcomes.

**Supplementary Information:**

The online version contains supplementary material available at 10.1186/s41687-022-00428-z.

## Plain English summary

Patient-reported outcomes (PROs) allow doctors and researchers to understand the patient perspective, such as how they are doing physically, mentally, or socially. When used, PROs can improve health and increase satisfaction of patients. Many clinics and hospitals are interested in using PROs in everyday care. Doctors, hospitals, and insurance companies are also using information from PROs to decide if the care they give is good quality. Unfortunately, certain groups of patients, such as racial and ethnic minorities and patients with low income, report worse PROs. Because of these differences, it will be important to make sure that PROs are being collected from all people, but not much is known regarding how this has been done. This study demonstrates what is known so far with regard to using PROs in everyday clinical care for these diverse patient groups. Findings from this study show that PROs can be successfully collected, but more work is needed in certain medical fields, and some types of patients have specific needs, concerns, or preferences with regard to PRO collection.

**Supplementary Information:**

The online version contains supplementary material available at 10.1186/s41687-022-00428-z.

## Background

Patient-reported outcomes (PROs) allow clinicians and researchers to better understand patients’ perceptions of their health, goals, treatment, and healthcare experiences across different domains, such as physical, mental, and social well-being [1, 2]. Patient-reported outcome measures (PROMs) are tools used to measure PROs in various disease contexts. Ideally PROMs are rigorously tested and validated. While many PROMs were developed for use in clinical trials [3], the use of PROMs in routine clinical care has become more widespread and has been shown to improve symptom control, supportive care measures, patient satisfaction, and health outcomes [4, 5]. In addition, there is increasing demand from healthcare payors, regulators, and administrators to incorporate PROs into routine clinical care for quality improvement and reimbursement purposes [6, 7].

Several logistical challenges regarding the implementation of PRO data collection into routine clinical practice have been noted, such as selection of appropriate and relevant PROMs, adequate staffing and data resources, patient compliance, and non-integration with electronic health records [6, 8–10]. However, an additional concern is ensuring equitable PRO implementation, such that PROs capture diverse and underrepresented patient populations [6, 11].

Given the current landscape of biomedical research, where less than 2% of 10,000 clinical trials included sufficient numbers of minority participants in one 2015 report [12], for example, it is unlikely that PRO implementation research would be immune to these issues of health equity and healthcare disparities in clinical research. PRO implementation in routine clinical care has been documented to have low adoption nationwide [13], which may further impede efforts to capture diverse populations. Additionally, reporting of certain factors such as race and ethnicity in clinical trials has not been widespread [14].

Routine PRO data collection is therefore likely predominant in White, higher socioeconomic, and English-speaking populations [15]. This is further supported by a systematic review of PRO implementation in routine care for patients with breast cancer, in which only 2 of 34 identified studies targeted minority patient populations [16] and a study of PRO implementation in integrative medicine, in which the majority of patients were White and had commercial insurance [17]. In addition, clinical PRO implementation studies have identified disparate response rates for many diverse and underrepresented populations across clinical specialties [18–21].

The lack of participation of certain patient populations in PRO data collection is concerning in light of a growing body of evidence demonstrating disparities in PROs among diverse and underrepresented patient populations. For example, PROs were significantly associated with race, education, and neighborhood poverty in a study of hip and knee arthroplasty [22], and lower income families experienced higher symptom burden and worse quality of life in a pediatric oncology study [23]. As the demand for PRO implementation in routine clinical care grows, it will therefore be critical to collect PROs from diverse and underrepresented patient populations to ensure representative sampling and reduce health disparities [24].

However, there is presently a limited understanding of the landscape of PRO implementation in these populations. This study therefore aims to review the literature to determine what is presently known regarding implementation of PROs in routine clinical care for diverse and underrepresented populations. The primary aim of this review is to characterize PRO implementation in routine clinical care, in terms of patient populations studied and clinical specialties that have evaluated PRO implementation. The secondary aims of this review are to analyze implementation outcomes and population-specific concerns, needs, and preferences regarding PRO collection. We believe that the review will advance our understanding of existing PRO programs and identify important areas of future research.

## Methods

Given the lack of knowledge regarding available evidence and breadth of coverage of PRO implementation in diverse and underrepresented populations across clinical specialties, a scoping review methodology was adopted for this study. Per guidance by Munn et al. [25], a scoping review enables determination of the studies available and overall focus of a research area, aligning with this study’s aims to identify the landscape of available studies, populations and clinical specialties represented, and implementation outcomes evaluated to date. The review was conducted in accordance with the Joanna Briggs Institute (JBI) methodology for scoping reviews [26].

### Data source and search

Studies were identified by searching the following electronic databases: MEDLINE (Ovid), Embase (Elsevier), Web of Science Core Collection (Clarivate Analytics), Cumulative Index to Nursing and Allied Health Literature (CINAHL: EBSCO), and PsycINFO (EBSCO). Searches were conducted between September 23 and 27, 2021. No date restriction was applied. The terms used to search each database were informed by previously published systematic reviews investigating PRO implementation in routine clinical care [16, 27, 28], as opposed to PROs used as evaluative assessments only for interventions in clinical trials, and relevant MeSH terms related to health disparities or diverse and underrepresented populations known to be affected by healthcare disparities [29]. The search was designed and conducted by a medical librarian with expertise in systematic and scoping reviews (PAB). Search strategy for the included databases is provided in the Additional file 1: Table S1.

### Study selection and eligibility

Studies were compiled into the Covidence (Veritas Health Innovation) citation manager. First, abstracts and titles were screened by three independent reviewers (CJH, RG, RD) using inclusion and exclusion criteria. Full texts were then reviewed by three independent reviewers (CJH, RG, RD) to determine whether studies met inclusion criteria. Disagreements at each phase were resolved through discussion among the three reviewers.

**Fig. 1:**
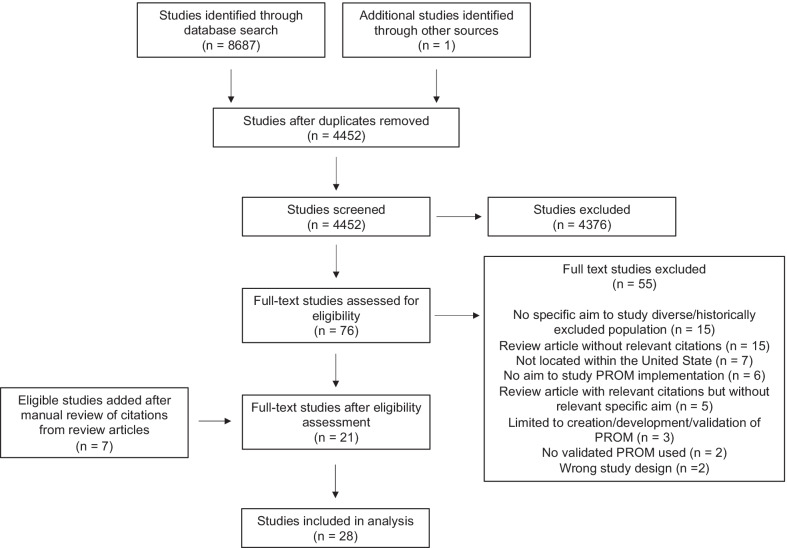
Preferred Reporting Items for Systematic Reviews and Meta-Analyses for Scoping Reviews (PRISMA-ScR) diagram detailing inclusion and exclusion of studies included in the analysis

Inclusion criteria were studies that (1) assessed PRO implementation in routine clinical care, (2) had a specific and explicit aim of studying PRO implementation in diverse or underrepresented populations, such as racial or ethnic minorities, sexual and gender minorities, elderly or geriatric populations ≥ 65 years of age, and populations with diverse literacy, educational, socioeconomic or geographic (rural or urban) status, (3) used a validated PROM, and (4) English language. Studies were limited to those conducted in the United States because diverse and underrepresented populations are in part defined by the specific historical, economic, and cultural contexts of their country of origin, as detailed in a previously published review [30]. In addition, certain sociodemographic factors such as insurance status and results concerning routine clinical care and PRO collection would be more generalizable to the unique healthcare system within the United States. Studies that focused on the development, creation, and/or validation of PROMs; use of PROMs as an outcome measure for some other primary intervention; and case reports, study protocols, editorials, dissertations, conference abstracts, and these were excluded. Citations within review articles from the search were manually reviewed to identify additional primary studies that met inclusion criteria. Results of the search as well as inclusion and exclusion of studies are reported according to the Preferred Reporting Items for Systematic Reviews and Meta-analyses for Scoping Reviews (PRISMA-ScR) flow diagram (Fig. 1) [31].

### Data analysis

Data from relevant studies were extracted into an a priori defined form by three reviewers (CJH, RG, RD), including study aims, sample size and demographics, geographic location of study settings, type of study setting, clinical specialty, validated PROM used, study limitations, if technology was used and type, and implementation outcomes. Implementation outcomes were categorized according to previously defined conceptual distinctions for implementation research: acceptability, adoption, appropriateness, costs, feasibility, fidelity, penetration, and sustainability [32]. These categorizations have been further refined for evaluation of technological interventions in healthcare [33] and have been used in previously published reviews of PRO implementation [28]. The quality and level of evidence (1–7) of studies were determined based on previously developed criteria by Melnyk et al., where level 1 corresponds to the highest quality evidence (systematic reviews, meta-analyses of randomized controlled trials, etc.) [34, 35].

## Results

### Study selection

The electronic database search yielded 8,687 records, and 1 record was identified through a review article known to study authors [16]. After duplicates were removed, the titles and abstracts of 4,452 records were each screened in duplicate according to pre-defined inclusion criteria (agreement > 95%). After screening these records, a total of 76 full-text articles were reviewed, of which 55 were excluded. Manual review of bibliographies from review articles identified in the search yielded an additional 7 studies that met inclusion criteria. A total of 28 studies were included in the analysis (Fig. 1).

### Quality assessment and study characteristics

The aims, quality, and general characteristics of studies are included in Table 1. Most studies were observational cohort studies with level 4 evidence (n = 21, 75%), followed by level 2 randomized controlled studies (n = 5, 17.9%), and level 6 qualitative studies (n = 2, 7.1%). Sample sizes within studies ranged from 10 [36] to 6454 [37]. Primary care was the most common specialty represented (n = 10, 35.7%) [11, 36, 38–45], followed by oncology (n = 4, 21.4%) [46–51], of which two studies were specific to breast oncology [46, 47] and two studies were specific to urologic/radiation oncology [49, 50]. Remaining specialties included rheumatology [52–55] (n = 4, 14.3%), psychology/psychiatry [56–58] (n = 3, 10.7%), and one study each (3.6%) for neurology [37], geriatrics [59], trauma [60], home health care [61], and orthopedic hand surgery [62].

**Table 1:** Characteristics of the studies (n = 28) included in the review

Study authors	Study aims	Study quality^a^	Sample size	Medical specialty	Study setting	Geographic location	PROM(s) used	PROM modality
Anderson et al. [46]	Refine and enhance a web-enabled app intervention that facilitates patient-provider communication about adjuvant endocrine therapy-related symptoms with a racially diverse sample of breast cancer survivors and healthcare providers	6: Qualitative study	39 (34 patients, 5 nurses)	Breast medical oncology	Network of fully integrated cancer care at 9 clinic locations	Tennessee, Arkansas, and Mississippi	Medication Adherence Reasons Scale Functional Assessment of Cancer Therapy-Endocrine Subscale (FACT-ES)—condensed version	THRIVE Intervention: web-enabled app with built-in, real-time alerts and EHR integration
Anderson et al. [47]	Assess the feasibility and efficacy of an automated pain intervention with PROs for underserved African American and Latina women	2: Randomized controlled trial	60	Breast medical oncology	Outpatient medical oncology clinic	Houston, TX	MD Anderson Symptom Inventory (MDASI) Barriers Questionnaire II (BQ-II)	Interactive voice response (IVR) system on a telephone; cell phone provided if patient needed
Arcia et al. [36]	Develop and test English- and Spanish-language tailored infographics of the Asthma Control Questionnaire and pulmonary function test results in a diverse population of adults with persistent asthma	4: Qualitative and cohort study	Phase I participatory design: 21 Phase II comprehension interviews: 10	Primary care	Federally qualified health center and primary care clinic	Philadelphia, PA; New York City, NY	Asthma Control Questionnaire (ACQ)	Pamphlets with infographics tailored to individual patient's ACQ score and PFT results
Calamia et al. [56]	Assess validity and feasibility of using a novel web-based application that employs self-administered approach for elderly patients to provide demographic data and self-assessments of self-rated health, depression, anxiety, and cognition	4: Cohort study	174	Psychology	Senior living community	Baton Rouge, LA	Face Name Hobby Recall (FNHR)—Immediate Free Recall, Immediate Recognition, Delayed Free Recall; Delayed Recognition; Grid Locations Immediate Recall (GLIR); Grid Locations Delayed Recall (GLDR); Symbol Line (SL); Visual Patterns (VP); Speeded Matching (SM); EQ-5D Visual Analog Scale (VAS); Logical Memory-Delayed Recall; Digital Symbol Coding (DSC); Free and Cued Selective Reminding Test (FCSRT); Geriatric Anxiety Inventory (GAI); Geriatric Depression Scale (GDS)	Novel web-based platform for self-reported demographic data and assessments of self-rated health, depression, anxiety, and cognition
Gabbard et al. [59]	Assess the feasibility of implementing an iPad-based symptom assessment tool in older adults with ESRD on hemodialysis	4: Cohort study	22	Geriatrics	Large academic tertiary medical center	North Carolina	Short-Form McGill Pain Questionnaire 2 (SF-MPQ-2); Patient Health Questionnaire-9 (PHQ-9); Generalized Anxiety Disorder 7 Item Survey (GAD-7); Dialysis symptom Index (DSI); Kidney Disease Quality of Life (KDQOL-36)	iPad application-delivery system for collecting electronic PROMs (ePROMs)
Gonzalez et al. [43]	Determine acceptability, administration times, and psychometric properties of an all-audio all-verbal speech-responsive depression screening questionnaire via cellular phone to English and Spanish speaking samples	2: Randomized controlled trial	52	Primary care	Health- and social-service facilities	San Diego, CA	Center for Epidemiological Studies—Depression scale (CES-D)	Computerized questionnaire implemented over the phone with voice recognition for interview responses, with option for touch-tone responses
Gonzalez et al. [44]	Study the reliability, validity, and acceptability of a bilingual computerized assessment of depression	4: Cohort study	166	Primary care	1 public research hospital 3 primary care clinics 1 mental health counseling center 1 social service agency	San Diego county, CA	Center for Epidemiological Studies—Depression scale (CES-D)	Computerized questionnaire implemented over the phone with voice recognition for interview responses
Hahn et al. [48]	Assess the feasibility of the implementation of a computerized QOL assessment tool among cancer patients with low literacy levels and computer skills	4: Cohort study	126	Oncology	3 cancer care centers	Chicago, IL	Functional Assessment of Cancer Therapy-General (FACT-G) Short Form-36 HealthSurvey (SF-36)	Touchscreen delivery method with multimedia components (visual, audio)
Hinami et al. [38]	Assess implementation and associated outcomes of an audio computer-assisted self interview technology in patients with low levels of literacy	4: Cohort study	1442	General internal medicine/primary care	General medicine clinic of a large, urban public healthcare system	Chicago, IL	National Institutes of Health Patient-Reported Outcomes Measurement Information System (PROMIS) [Global Physical Health; Global Mental Health]; Memorial Symptom Assessment Scale (MSAS); Patient Health Questionnaire 2-item (PHQ-2) short form	Touch-screen enabled audio computer-assisted self-interview (ACASI) software in English or Spanish
Hirsh et al. [55]	Evaluate vulnerable patients' attitudes regarding PGA-VAS implementation in a safety-net rheumatology clinic	4: Cohort study	300	Rheumatology	Safety-net hospital clinic	Denver, CO	Visual analog scale patient global assessments (PGA-VAS): disease activity score-28 (DAS28-PGA-VAS) and multidimensional health assessment questionnaire (MDHAQ-PGA-VAS)	Written questionnaires
Jacoby et al. [60]	Determine the accessibility and feasibility of mobile health monitoring for long-term outcomes in a population of trauma patients with barriers to health and social care	4: Qualitative and cohort study	25	Trauma	Level 1 trauma Center	Philadelphia, PA	Patient-Reported Outcomes Measurement Information System (PROMIS) Sleep Disturbance Short Form; Brief Pain Inventory	Mobile health technology (FitBit) with web-based questionnaire platform
Jiwani et al. [39]	Assess the feasibility of using PROMIS questionnaires in routine diabetes care for a historically under-resourced/underserved population	2: Randomized controlled trial	26	Primary care	Community health center	Harris County, TX	Patient-Reported Outcome Measurement Information System (PROMIS): PROMIS-57 and PROMIS-Global Health (GH)	M-Health technology (mobile health)
Kasturi et al. [52]	Assess the feasibility of administering PROMIS computerized adaptive tests to a diverse cohort of patients with systemic lupus erythematosus	4: Cohort study	204	Rheumatology	Lupus center within a hospital	New York, NY	14 PROMIS CATs: Physical Function (v1.2), Mobility (v1.2), Pain Behavior (v1.0), Pain Interference (v1.1), Ability to Participate in Social Roles (v2.0), Satisfaction with Social Roles and Activities (v2.0), Fatigue (v.1.0), Sleep Disturbance (v1.0), Sleep-Related Impairment (v1.0), Applied Cognition-Abilities (v1.0), Applied Cognition-General Concerns (v1.0), Anger (v1.1), Anxiety (v1.0), and Depression (v1.0) 36-Item Short Form Health Survey (SF-36) and LupusQoL-US	PROMIS CAT administered either on site (desktop, tablet, or smartphone) or remotely using a device of patient's choice
Lapin et al. [37]	Determine patient experience of PROM collection, with a specific aim at assessing subgroups with historically lower reported quality of care	4: Cohort study	6454	Neurology	15 ambulatory neurology centers	Ohio	10-item Patient-Reported Outcome Measurement Information System Global Health short form (PROMIS-GH); Patient Health Questionnaire-9 (PHQ-9)	PROMs completed on electronic tablet before their visit or at home via their patient portal
Liu et al. [53]	Assess perspectives on PRO data visualization in a diverse population of rheumatology patients via patient and clinician focus groups	6: Qualitative study	25	Rheumatology	University or county based rheumatology clinic	California	Clinical Disease Activity Index (CDAI); Patient-Reported Outcomes Measurement Information System-physical function (PROMIS-PF); unspecified pain score	PRO data visualization dashboards that incorporated patient feedback
Loo et al. [40]	Assess implementation of an electronic PRO system in an urban community clinic that serves a diverse population, of which > 50% are LGBTQ	4: Cohort study	n/a	Primary care	3 primary care clinics	Boston, MA	Patient Health Questionnaire-9 (PHQ-9); PHQ-9 modified for adolescents (PHQ-A) Alcohol Use Disorders Identification Test (AUDIT-C), learning needs assessment, smoking and tobacco, fall risk assessment, intimate partner violence, Drug Abuse Screening Test-10 (DAST-10), Generalized Anxiety DIsorder-7 (GAD-7), Edinburgh postpartum screen	Tablet device containing PROs given at clinic visits by medical assistants and completed in the waiting room
Munoz et al. [45]	Determine the reliability and acceptability of a computerized depression screening measure for underserved Spanish-speaking patients	2: Randomized controlled trial	38	Primary care	Public sector primary care depression clinic	San Francisco, CA	Center for Epidemiological Studies—Depression scale (CES-D)	Computerized questionnaire with voice recognition for interview responses
Nyirenda et al. [61]	Evaluate the feasibility of implementing PROMs in a home health care setting of predominately older adults	4: Cohort study	91	Home health care	2 home health care agencies	n/a	Patient-Reported Outcomes Measurement Information System (PROMIS)	Tablet with electronic PROMIS survey
Ramsey et al. [57]	Assess the perceived acceptability, adherence rates, and reasons for nonadherence to smartphone-based ecological momentary assessment among older patients	4: Cohort study	103	Psychiatry	n/a	Greater San Diego, CA; Greater St. Louis, MO	Ecological momentary assessment (EMA)	Smartphone-based EMA assessment
Samuel et al. [49]	Assess ePRO user experiences and perceived valued among a diverse patient population	4: Cohort study	79	Urologic oncology; radiation oncology	Hospital urology and radiation oncology clinics	North Carolina	Patient-Reported Outcomes Measurement Information System-short form (PROMIS-SF) Bladder Cancer Index Expanded Prostate Cancer Index Composite	ePROs completed at home or in clinic using a web-based or automated telephone system Patients and clinicians received a symptoms summary report at each visit
Sarkar et al. [42]	Assess usability of mobile applications for diabetes, depression, and caregiving among a diverse and vulnerable patient populations	4: Cohort study	26	Primary care	Urban outpatient primary care clinic	San Francisco, CA	Patient Health Questionnaire-9 (PHQ-9)	11 mobile applications available for iPhones or Androids, one of which involved PHQ-9 data entry
Scholle et al. [11]	Evaluate factors associated with PROM implementation in routine clinical are for a diverse patient population	4: Cohort study	490	Primary care	2 primary care clinics (FQHC and academic medical center)	n/a	Patient-Reported Outcomes Measurement Information System-29 (v2.0)	Generally completed on paper or read out loud by staff if requested/by phone
Shipp et al. [62]	Evaluate patient use of an ePRO system, with a specific aim of identifying patterns in subgroups of underrepresented populations	4: Cohort study	4898	Orthopedics (hand)	Specialty hand center	Baltimore, MD	Unspecified	Web-based intake platform that incorporates PROs
Smith et al. [50]	Assess feasibility of enrollment and collecting PRO data in routine urologic care for a racially diverse cohort	4: Cohort study	76	Urologic oncology; radiation oncology	Genitourinary oncology clinics	North Carolina	Patient-Reported Outcomes Measurement Information System (PROMIS) Sleep Disturbance, Fatigue, Anxiety, Depression, Constipation, Diarrhea, Sexual Function, and Satisfaction v1.0 Expanded Prostate Cancer Index Composite (EPIC) Urinary Domain Bladder Cancer Index Urinary Domain	Tablet-based PRO survey in clinic with option to complete at home via the web or an automated phone survey
Stonbraker et al. [41]	Design symptom reports from longitudinal PRO data for end users, with an aim of understanding needs of patients with low health literacy	4: Cohort study	55	HIV primary care	Private office	New York, NY	Symptom Burden Score (expanded version of the 20-item HIV symptom index)	Use of the VIP-HANA mobile phone app, which incorporates PROs and symptom self-management strategies as well as longitudinal symptom reports
Wahl et al. [54]	Determine the feasibility of implementing the PF-10a in a racially and ethnically diverse population with rheumatoid arthritis	4: Cohort study	326	Rheumatology	Hospital rheumatology clinic	San Francisco, CA	Patient-Reported Outcomes Measurement Information System-Physical Function-10a (PROMIS-PF-10a) Health Assessment Questionnaire Disability Index (HAQ) Clinical Disease Activity Index (CDAI)	Patients complete survey in waiting room and the MA enters responses into the EHR
Wolford et al. [58]	Compare computer-assisted interviewing (CAI) with face-to-face assessments for people with severe mental illness known to be impacted by literacy and concentration problems	2: Randomized controlled trial	245	Psychiatry	Acute inpatient and outpatient publicly funded service settings	North Carolina, Maryland, and New Hampshire	PTSD Checklist (PTSD) Dartmouth Assessment of Lifestyle Instrument (DALI) AIDS Risk Inventory (ARI)	Web-based platform for questionnaire completion, with large text and speech options, with the opportunity to repeat questions
Zullig et al. [51]	Assess feasibility and effectiveness of PRO collection in a rural, underserved geriatric cancer clinic	4: Cohort study	44	Medical oncology	Oncology clinics	Laurinburg and Lumberton, North Carolina	Senior Adult Oncology Program (SAOP) screener	Instrument administered by clinic nurse or medical oncologist and answers uploaded into EHR

^a^Study quality was assessed according to Levels of Evidence (1–7) previously described by Melnyk et al. [34]

App, application; EHR, electronic health record; PRO, patient-reported outcome; PROM, patient-reported outcome measure; ePRO, electronic patient-reported outcome; ePROM, electronic patient-reported outcome measure; IVR, interactive voice response; PFT, pulmonary function test; ESRD, end-stage renal disease; QOL, quality of life; ACASI, audio computer-assisted self interview; PROMIS CAT, patient-reported outcomes measurement information system computer adaptive test; LGBTQ, lesbian gay bisexual, transgender, queer; VIP-HANA, video information provider-HIV-associated non-AIDS; MA, medical assistant; CAI, computer-assisted interviewing

The demographics and characteristics of study populations are detailed in Table 2. Most included a study population that was majority female (n = 19, 67.9%) compared to majority male (n = 3, 10.7%). Some studies reported multiple samples with variable gender majorities (n = 4, 14.3%) or did not report gender (n = 2, 7.1%). Most studies (n = 15, 53.6%) included majority non-White populations and reported educational attainment and/or literacy (n = 19, 67.9%). In contrast, most studies did not report income or employment status (n = 17, 60.7%) or insurance status (n = 19, 67.9%). For studies that reported participants’ age, the age range among studies was 26.7 to 75.9 years. The majority of studies (n = 23, 82.1%) used electronic PRO (ePRO) data collection.

**Table 2:** Study population demographics and characteristics

Study authors	Mean age (years)	Gender	Race/ethnicity	Education/literacy	Income/employment status	Insurance status	Other
Anderson et al. [46]	64	Sample in single-race focus groups: 100% female	Sample in single-race focus groups: 46% Black 54% White	n/a	n/a	n/a	n/a
Anderson et al. [47]	Intervention: 49.6 Control: 50.5	100% female	Intervention: 42% African American 58% Latina Control: 41% African American 59% Latina	Intervention: 10.6 years of education Control: 10.0 years of education	Intervention 52% unemployed 6% employed Control: 52% unemployed 14% employed	n/a	Intervention: 48% married 52% unmarried Control: 45% married 55% unmarried
Arcia et al. [36]	Phase I: 50.2 Phase II: 42.2	Phase I: 71% female 29% male Phase II: 70% female 30% male	Phase I: 62% non-Hispanic Black 38% Hispanic Phase II: 70% non-Hispanic Black 20% Hispanic 10% refused	Phase I: 24% some high school 38% high school diploma 33% some college 5% Bachelor's Phase II 10% some high school 30% high school diploma 20% some college 10% Associate's 20% Bachelor's 10% missing	Mean income per person in household per year: Phase I: $9,789 Phase II: $9,240	Phase I: 48% Medicaid 10% Military/VA 24% commercial 19% missing Phase II: 100% Medicaid	n/a
Calamia et al. [56]	MMSE ≥ 25: 71.64 MMSE ≤ 25: 75.94	MMSE ≥ 25: 71.6% female 28.4% male MMSE ≤ 25: 31.6% female 68.4% male	MMSE ≥ 25: 90.3% White 4.5% African American 1.3% Bi-racial 0.01% Native American 3.89% missing MMSE ≤ 25: 89.5% White 5.3% African American 5.2% missing	MMSE ≥ 25: 6.5% GED 21.3% some college 3.9% Associate's 26.5% Bachelor's 33.5% Master's 3.9% Doctorate 4.4% missing MMSE ≤ 25: 5.3% GED 5.3% some college 5.3% Associate's 36.8% Bachelor's 21.1% Master's 10.5% Doctorate 15.7% missing	n/a	n/a	n/a
Gabbard et al. [59]	69.4	63.6% female 36.4% male	81.8% Black/African American 13.6% White 4.6% Asian	27.3% less than high school graduate 22.7% high school graduate or equivalent 18.2% some college or tech/vocational 9.1% Master's Degree 4.6% Professional Degree	Annual household income: 63.6% < $20,000 13.6% $20,000-$40,000 22.7% > $40,000–75,000 0% > $70,000	n/a	4.6% single, never married 31.8% married 31.8% divorced 27.3% widowed 4.6% separated
Gonzalez et al. [43]	Spanish-speaking: 26.7 English-speaking: 36.6	Spanish-speaking: 70% female 30% male English-speaking: 54% female 46% male	Spanish-speaking: 97% Latino (83% Mexican, 4% Nicaraguan, 13% n/a) English-speaking: 82% White 9% African American 9% other	Spanish-speaking: 7.1 years of education English-speaking: 12.9 years of education	n/a	n/a	n/a
Gonzalez et al. [44]	Spanish-speaking: 35.2 English-speaking: 47.4	Spanish-speaking: 77% female 23% male English-speaking: 42% female 58% male	Spanish-speaking: 99% Latino (95% Mexican), 1% European American English-speaking: 79% European American 11% African American 4% American Indian, 4% Latino 1% Asian 1% Jewish	Spanish-speaking: 10.9 years of education English-speaking: 13.7 years of education	Unspecified, but no difference between groups	n/a	n/a
Hahn et al. [48]	50.9	69.8% female 30.2% male	55.5% Black/African-American 12.7% Hispanic/Latino 29.4% White, non-Hispanic 1.6% Other 0.8% Asian or Pacific Islander	Education Levels: 5.6% 0–6th Grade 6.3% 7th–8th Grade 27.8% some high school 34.9% high school grad/GED 15.1% some college 10.3% college degree 50.8% low literacy level (7th-grade reading comprehension) 39.7% high literacy 9.5% pending	n/a	n/a	Previous computer experience: 38.9% none 14.3% a few times/year 24.6% every month/week 19.8% almost every day 2.4% unknown
Hinami et al. [38]	57	58% female 42% male	53% non-Hispanic Black 24% Hispanic 10% non-Hispanic White 6% non-Hispanic Asian 7% other	n/a	n/a	n/a	20% preferred to complete the survey in Spanish
Hirsh et al. [55]	53	77% female 23% male	n/a	64% completed high school or less 18% inadequate health literacy 10% marginal health literacy	72.8% below the federal poverty line (< $15,000/year)	n/a	72% English-speaking
Jacoby et al. [60]	40.2	100% male	96% African American 4% Native American	8% some high school 60% high school graduate or GED 20% some college 8% college graduate 4% trade/technical training	36% < $10,000 32% $10,000–29,999 24% $30,000–49,999 4% $50,000–79,000 4% missing	20% private insurance 4% Medicare 32% Medicaid 32% self-pay/uninsured 12% missing	n/a
Jiwani et al. [39]	54.7	57% female 43% male	68% African American	n/a	n/a	n/a	n/a
Kasturi et al. [52]	40.5	92.9% female 7.1% male	37.7% White 29.9% Black 12.8% Asian 19.6% other 28.4% Hispanic/Latino	16.7% high school or less 24.1% some college 59.1% college or beyond	n/a	35.8% Medicaid 10.3% Medicare 53.9% private	33% on disability
Lapin et al. [37]	Sample that completed satisfaction survey: 57.7	Sample that completed satisfaction survey: 59.2% female 40.8% male	Sample that completed satisfaction survey: 8.5% non-white	Sample that completed satisfaction survey: 47.3% college graduate 33% some college 18.3% high school graduate 1.4% less than high school	Sample that completed satisfaction survey: Median income $54,200	n/a	Sample that completed satisfaction survey: 70.5% married
Liu et al. [53]	59.9	80% female 20% male	n/a	64% adequate health literacy 36% limited health literacy	n/a	n/a	60% English-speaking 40% Spanish-speaking
Loo et al. [40]	n/a	n/a	unspecified but clinic comprised of 30% racial/ethnic minorities	n/a	n/a	n/a	over 50% of clinic population identifies as LGBTQ
Munoz et al. [45]	Spanish-speaking: 50.3 English-speaking: 51.9	Spanish-speaking: 68.4% female 31.6% male English-speaking: 42.1% female 57.9% male	Spanish-speaking: 100% Latino English-speaking: 63.1% White 31.6% African American 5.3% Asian American	Spanish-speaking: 10.2 years of education English-speaking: 12.6 years of education	n/a	n/a	Spanish-speaking: 74% had novice computer experience
Nyirenda et al. [61]	71.9	64% female 33% male 3% unknown	87% White 92% non-Hispanic	28.1% did not complete high school 71.9% at least high school 28.1% some college 9.3% associate degree or higher	n/a	n/a	n/a
Ramsey et al. [57]	71.9	73% female 27% male	81% White 9% African American 5% Asian/Pacific Islander 4% Hispanic 2% Other/Unknown	15.6 years	n/a	n/a	n/a
Samuel et al. [49]	Black: 62.8 White: 66.8	Black: 16.7% female 83.3% male White: 10.2% female 89.8% male	38% Black 62% White	Black: 33.3% high school or less 30% some technical college 16.7% some college 10% college 10% graduate White: 18.4% high school or less 20.4% some technical college 20.4% some college 14.3% college 26.5% graduate	Black: 23.3% employed 6.7% unemployed White: 42.9% employed 0% unemployed	Black: 26.7% private insurance 80% public insurance White: 61.2% private insurance 65.3% public	n/a
Sarkar et al. [42]	57	69% female 31% male	58% Black or African American 8% Hispanic/Latino 8% Asian/Pacific Islander 27% White	69% limited health literacy 31% adequate health literacy	Reportedly low income, but no specific data	100% Medicare/Medicaid or no insurance	85% use a computer 38% use a cell phone 50% use a smartphone 31% use a tablet 4% have no device
Scholle et al. [11]	Site 1: 27.3% 18–64 22.7% 65 or older Site 2: 28.8% 18–64 21.7% 65 or older	Site 1: 28.2% female; 24.2% male Site 2: 27.1% female 25% male	Site 1: 29.3% White 31.3% Black 25.4% Hispanic Site 2: 26.3% White 32.5% Black 16.5% Hispanic	n/a	n/a	Site 1: 30.2% commercial insurance 26.5% public 17.1% uninsured Site 2: 20.6% commercial insurance 29.2% public 21.5% uninsured	Site 1: 24.1% preferred Spanish
Shipp et al. [62]	n/a	n/a	n/a	n/a	n/a	n/a	n/a
Smith et al. [50]	66 (median)	13% female 87% male	38% Black 62% White	25% high school or less 29% vocational school 25% college 21% graduate	36% full or part-time employed 7% medical leave or unemployed 57% retired	12% Medicaid 41% Medicare 19% Medicare with supplemental 27% private	76% married, living with partner
Stonbraker et al. [41]	55.4	63.6% female 36.4% male	63% African American/Black 24.1% Hispanic/Latino 7.4% White 5.6% other	40% less than high school 40% completed high school 20% more than high school 89.1% likely limited health literacy 10.9% adequate health literacy 91% likely limited graph literacy 9% adequate graph literacy	n/a	n/a	81.8% English-speaking 18.2% Spanish-speaking
Wahl et al. [54]	59	81.6% female 19.4% male	48% White 8% African American 15% Hispanic 18% Asian 10% Other	n/a	n/a	52% private insurance 36% Medicare 11% Medicaid	82% preferred English language
Wolford et al. [58]	Group 1 (computer/computer): 42.7 Group 2 (computer/person): 41.1 Group 3 (person/computer): 42.4 Group 4 (person/person): 41.8	Group 1 (computer/computer): 53% female 47% male Group 2 (computer/person): 39% female 61% male Group 3 (person/computer): 47% female 53% male Group 4 (person/person): 46% female 54% male	Group 1 (computer/computer): 74% White 15% African American 11% Native American Group 2 (computer/person): 68% White 23% African American 9% Native American Group 3 (person/computer): 61% White 32% African American 7% Native American Group 4 (person/person): 69% White 25% African American 3% Hispanic 3% Native American	n/a	n/a	n/a	All groups with some percentage of patients with schizophrenia, schizoaffective disorder, bipolar disorder, and/or major depression
Zullig et al. [51]	71.5	65.9% female 34.1% male	38.6% White 36.4% Black 25.0% American Indian	n/a	unspecified, but clinics serve population with 20% living at or below the federal poverty level	20.5% commercial insurance 79.5% Medicare/Medicaid/VA 0% uninsured	47.7% married/living with partner 9.1% single/never married 18.2% divorced/separated 25.0% widowed

VA, veterans affairs; MMSE, mini-mental state exam; GED, general educational development; LGBTQ, lesbian gay bisexual transgender queer

### Implementation outcomes

Implementation outcomes are detailed in Table 3. Most studies evaluated feasibility (n = 27, 96.4%), followed by acceptability (n = 19, 67.9%), adoption (n = 18, 64.3%), and appropriateness (n = 10, 35.7%). Four studies assessed fidelity (14.3%) and three studies assessed costs (10.7%). One study each evaluated for penetration (3.6%) and sustainability (3.6%). Specific details and examples of each implementation outcome are detailed in Additional file 2: Table S2. Of those assessing feasibility, the majority assessed PRO completion rates over time (n = 17, 60.7%). Fewer assessed time needed to complete PRO assessments (n = 7, 25%) or impact on clinic workflows, such as the need for staff assistance in PRO completion or work burden (n = 7, 25%). Of those assessing adoption, few reported patients’ intention to try the studied PRO collection method again or in another setting (n = 3, 10.7%) and one reported clinicians’ intention to adopt new PRO technology in practice [53]. Of the three studies that assessed costs, none assessed cost-effectiveness. With regard to the one study assessing penetration and sustainability, the number of providers interfacing with the PRO system increased over time and the program was able to be maintained over a 5-year study period [40].

**Table 3:** Implementation outcomes of studies classified according to Proctor et al. [32] taxonomy

Study authors	Acceptability	Adoption	Appropriateness	Costs	Feasibility	Fidelity	Penetration	Sustainability
Anderson et al. [46]	X		X		X			
Anderson et al. [47]		X			X			
Arcia et al. [36]	X		X		X			
Calamia et al. [56]					X	X		
Gabbard et al. [59]	X	X			X			
Gonzalez et al. [43]	X				X			
Gonzalez et al. [44]	X							
Hahn et al. [48]		X		X	X			
Hinami et al. [38]		X			X			
Hirsh et al. [55]	X				X			
Jacoby et al. [60]	X	X	X		X			
Jiwani et al. [39]		X			X			
Kasturi et al. [52]	X	X	X		X			
Lapin et al. [37]	X		X		X			
Liu et al. [53]	X	X	X		X	X		
Loo et al. [40]	X	X		X	X		X	X
Munoz et al. [45]	X				X	X		
Nyirenda et al. [61]	X	X	X		X			
Ramsey et al. [57]	X	X	X		X			
Samuel et al. [49]	X	X	X		X			
Sarkar et al. [42]	X				X			
Scholle et al. [11]	X	X	X		X			
Shipp et al. [62]		X			X			
Smith et al. [50]		X			X			
Stonbraker et al. [41]	X				X	X		
Wahl et al. [54]		X			X			
Wolford et al. [58]	X	X		X	X			
Zullig et al. [51]		X			X			

### Overall concerns, needs, and preferences of populations studied

Overall concerns, needs, and preferences were abstracted from studies and detailed in Table 4. Among the concerns were disparities in PRO completion among racial and ethnic minorities [11, 38, 50, 62], Spanish-speaking patients [11], populations with low income/employment status and low educational or health literacy [62], and elderly or geriatric populations [11, 38, 62]. Needs among populations included additional help in completing surveys among patients with low income/education [55] and a suggestion for a tutorial video for ePRO use among elderly patients [59]. Preferences among populations included a higher likelihood of Black patients selecting automated telephone over web-based surveys [49, 50], conflicting results of Spanish-speaking patients preferring face-to-face interviews vs. electronic data collection [43–45], symptom report display using bar graphs with “emojis” for low health literacy, majority Black patients [41], and preference for using a finger over stylus for tablet-based PRO collection in elderly patients [61].

**Table 4:** Overall concerns, needs, and preferences of populations identified in the review

Population description
*Racial and ethnic minority populations*
Concerns: Non-Hispanic Black patients less likely than Hispanic, non-Hispanic White, and non-Hispanic Asian patients to be able to complete a touch-screen enabled computer-assisted self-interview [38] Non-Hispanic black patients more likely and Hispanic patients who preferred Spanish less likely to complete PROMs than Non-Hispanic White patients who speak English [11] Late responses to web-based PRO platform associated with racial/ethnic minorites [62] Black patients less likely than White patients to complete tablet-based PRO [50] Low income, limited health literacy, majority Black patients had more difficulty with PRO data retrieval than data completion within commercially available mobile apps on iPhones and Androids [42] Needs: n/a Preferences: No race-based preferences for web-based app content or features [46] Black patients were more likely than White patients to select automated telephone surveys, although web-based delivery was most common overall; Black patients had greater difficulty understanding questions and the summary report than White patients [49] Higher proportion of Black vs. White patients preferred telephone-based survey formats [50] Other: Mobile health PROs and health monitoring successful among low income, majority Black trauma survivors [60] Mobile health PROs successful among rural, traditionally underserved, majority Black patients with diabetes [39] Non-White patients were more satisfied than White patients with their care as a result of PROM collection [37] Interactive voice response system deemed feasible and improved symptom severity among majority unemployed African American and Latina patients [47]
*Non-English-speaking populations*
Concerns: Spanish-language groups found longitudinal PRO outcome data more difficult to understand than English-language groups [53] Hispanic patients who preferred Spanish less likely than Hispanic patients who preferred English to complete PROMs [11] Needs: Spanish-language groups did not anticipate challenges using a dashboard with an interpreter [53] Preferences: Infographics well-received and comprehended by English- and Spanish-speaking populations [36] Spanish-speaking population less likely to prefer a computer-telephone-based PRO method than English-speaking population [43] The majority of Spanish-speakers preferred face-to-face interviewing [44] Other: n/a
*Populations with low income/employment status*
Concerns: Late responses to web-based PRO platform associated with low income [62] Half of a low SES, low education population found PROs confusing [55] Low income, limited health literacy, majority Black patients had more difficulty with PRO data retrieval than data completion within commercially available mobile apps on iPhones and Androids [42] Needs: Half of a low SES, low education population wanted help completing surveys [55] Preferences: n/a Other: Low income patients had more favorable experiences with PROM collection than patients within the top 3 quartiles of income [37] PRO collection with EHR upload feasible in a low-resource clinical setting with a 25% Native American population [51] Interactive voice response symptom deemed feasible and improved symptom severity among majority unemployed African American and Latina patients [47]
*Populations with low educational or health literacy*
Concerns: Low health literacy patients more likely to find PRO outcome dashboard and longitudinal data difficult to understand than patients with higher health literacy [53] Late responses to web-based PRO platform associated with lower education [62] Low income, limited health literacy, majority Black patients had more difficulty with PRO data retrieval than data completion within commercially available mobile apps on iPhones and Androids [42] Needs: n/a Preferences: A bar graph combined with emojis was the most preferred PRO symptom display format among low health literacy, majority Black patients [41] Computer-based interview was preferred over in-person interview for patients with psychiatric disorders known to be impacted by low literacy [58] Other: Completion rates and time needed to complete surveys on a touchscreen-based display similar between patients with low and high literacy [48]
*Elderly and/or geriatric populations*
Concerns: Older patients less likely than younger patients (mean 57) to be able to complete a touch-screen enabled computer-assisted self-interview [38] Older patients less likely than patients aged 18–64 to complete PROMs [11] Late responses to web-based PRO platform associated with older age [62] Among elderly patients using smartphone-based PROs, there was a discrepancy between perceived vs. actual survey completion adherence [57] Needs: Self-administered web-based collection system for elderly patients required little assistance from staff [56] Older, majority Black, low-income hemodialysis patients found iPad-based PROs easy to use, but desired a tutorial video [59] Preferences: Older patients in home health care settings found tablet-based PRO collection easy to use with a preference for using finger over stylus [61] Other: Smartphone-based PROs successful among elderly patients [57]
*Sexual and gender minority populations*
Concerns: n/a Needs: n/a Preferences: ePRO collection was appreciated by a > 50% LGBTQ clinic population [40] Other: ePRO collection made participants of a > 50% LGBTQ clinic population feel that they more direct participants in their care [40]

## Discussion

This review synthesizes studies to date that have explicitly aimed to evaluate PRO implementation in routine clinical care for diverse and underrepresented patient populations in the United States across all clinical specialties, thereby taking an important step in furthering our understanding in this area. Given growing demands for routine clinical PRO collection, this review specifically evaluated study quality, demographics, implementation outcomes, and patient perspectives in order to inform existing and emerging PRO programs as well as to identify areas requiring further research.

This review demonstrated that relatively few high-quality studies such as randomized-controlled trials have been conducted. In addition, studies are not representative of all clinical specialties and skew predominately toward primary care settings. In particular, there was a paucity of studies conducted in surgical, obstetric, and pediatric settings. While this may be partly explained by pre-existing disparities in access to and quality of care in these settings [63–66], it will be important to study PRO implementation across the entirety of the healthcare spectrum with particular attention to those presently unrepresented in research to date.

In recent years, several health information technology interventions have been developed to better facilitate electronic PRO (ePRO) implementation, such as web-based platforms, tablets, and mobile applications. While concerns have been raised, given that there are disparities in smartphone and tablet computer ownership as well as internet access [67], our study highlights that ePRO collection is widely acceptable and feasible among diverse and underrepresented patient populations. While some studies suggested that face-to-face PRO collection may be preferred by certain populations (e.g., primarily Spanish-speaking individuals [43, 44]), another study conducted in a similar population demonstrated preference for computerized data collection [45]. Moreover, these studies were published over 20 years ago and may not be representative of populations today that have had more time to adjust to new technologies. This is supported by recent research showing that reliance on smartphones for internet access has become increasingly more common among Americans with lower socioeconomic status as well as racial and ethnic minority populations [67]. However, relying on ePROs alone may not be sufficient to maintain equitable PRO collection, as evidenced by one report demonstrating profound racial and ethnic disparities when transitioning from tablet-based to web-based PRO collection [68]. Our study highlights several key findings regarding the unique needs and concerns of populations in various clinical settings using ePROs, such as inclusion of bar graphs with “emojis” for symptom reports [41] or touchscreens with visual and audio components, for example [48]. This not only emphasizes the need for further research in these populations, but also suggests that programs would likely benefit from specifically tailoring ePROs to the populations they serve.

Notably, the least studied implementation outcomes in studies to date were penetration and sustainability. While it is still helpful to understand elements of PRO implementation such as acceptability and feasibility in the short-term, it is evident that less is known regarding longer-term and institution-wide outcomes of these interventions. This is problematic, given that PRO implementation programs can require additional staffing and data resources [8, 10], which may be prohibitive for low-resourced settings where many diverse and underrepresented populations receive care, especially outside of short-term, intensive study settings. As evidenced by the 5-year study period in the one study in this review with sustainability and penetration outcomes [40], another potential barrier to studying these outcomes is the time needed for longer, prospective studies. Bolstering partnerships between higher-resourced academic centers with existing PRO programs and lower-resourced settings may therefore provide one solution.

Moreover, cost was another infrequently assessed outcome. Consequently, cost-effectiveness data of PRO implementation was largely missing. Alongside staffing and data resource concerns, the cost of PRO implementation may preclude implementation in resource-constrained settings where many diverse and underrepresented populations receive care. While one study reported on the ability to maintain long-term costs [40], the financial requirements and impacts of PRO implementation for diverse and underrepresented populations remains largely to be determined. Nonetheless, the reduction of healthcare disparities itself has been estimated to substantially reduce healthcare spending [69, 70]. With the aim of reducing disparities through these implementation programs, cost data will also be helpful in further characterizing the cost-effectiveness and healthcare value of routine PRO collection.

While the studies within this analysis elucidated important findings within underrepresented patient populations, it is important to note that more work is needed to elucidate which specific determinants of health within populations drive certain outcomes as well as the way in which identities and social determinants intersect [71]. Most studies, for example, reported racial and ethnic demographics of their study sample, however much fewer reported income, employment, and insurance status. This is problematic, given that racial or ethnic minority outcomes may be confounded by other unreported social factors, such as low income or unemployment. In addition, results and data analysis were often stratified by race or ethnic minority status alone, without delving deeper into the specific social determinants of health (i.e. transportation, perceived and actual racism, work environments). As such, future work evaluating implementation within these populations should more deeply investigate how social determinants of health drive disparate acceptability, adoption, or feasibility outcomes, for example.

Our study had some limitations. First, studies were limited to the United States in order to increase generalizability of results, given that diverse and underrepresented populations are defined in part by the history, demographics, culture, and economy of their country of origin. Although international studies such as those investigating routine PRO collection in rural Australia [72], use of robots for routine PRO collection among elderly adults in the Netherlands [73], and ePRO collection among a diverse, urban clinic population living with HIV in Canada [74] may have relevant and generalizable findings for clinical environments in the United States, we deemed these beyond the scope of this review. However, the narrower scope of this review allowed for greater specificity of findings for certain populations, such as Black Americans and non-English-speaking populations, within the unique context of PRO programs in the United States. Second, we only included studies that had a specific and explicit aim of studying PRO implementation in a diverse and/or underrepresented population. There is a possibility that studies investigating clinical PRO implementation more broadly had potentially relevant incidental findings for these populations, however this review importantly highlights studies that fill critical gaps in the literature by intentionally aiming to study these populations. In addition, this review studied diverse populations known to be affected by healthcare disparities in the United States. Third, our study focused on the implementation of already developed and validated PROMs. Addressing disparities in PRO data collection will also require equitable PROM development, testing, and validation within diverse and underrepresented populations before implementation occurs [15].

## Conclusions

As existing healthcare systems expand and new systems develop PRO data collection programs, it will be imperative to ensure that PRO data collection is not only representative of all patients but also equitable in its implementation in routine clinical care [15]. While this study highlights several important considerations for PRO implementation in diverse and underrepresented populations, it simultaneously calls attention to the paucity of research in this area to date. Future studies of PRO implementation will be needed across the healthcare spectrum in order to address existing disparities and promote health equity alike.

## Supplementary Information

**Additional file 1: Table S1**. Search strategy by databases included in the review.

**Additional file 2: Table S2**. Details of the specific implementation outcomes by studies included in the review.

## Data Availability

The datasets used and/or analyzed during the current study are available from the corresponding author on reasonable request.

## Abbreviations

PRO: Patient-reported outcome
PROM: Patient-reported outcome measure
CINAHL: Cumulative Index to Nursing and Allied Health Literature
ePRO: Electronic patient-reported outcome
PRISMA-ScR: Preferred Reporting Items for Systematic Reviews and Meta-analyses for Scoping Reviews
