# Supplementary material for: Implementing patient-reported outcomes in routine clinical care for diverse and underrepresented patients in the United States
Source: J Patient Rep Outcomes. 2022 Mar 7;6:20. doi: 10.1186/s41687-022-00428-z (PMC8901833; doi:10.1186/s41687-022-00428-z)
Supplement: Supplementary file 1 — Additional file 1: Table S1. Search strategy by databases included in the review. [file 41687_2022_428_MOESM1_ESM.docx]

**Additional file 1: Table S1.** Search strategy by databases included in the review

| **MEDLINE (Ovid)**  **Ovid MEDLINE(R) and Epub Ahead of Print, In-Process, In-Data-Review & Other Non-Indexed Citations, Daily and Versions(R) 1946 to September 22, 2021**  **Search Date: September 23, 2021**  **2,282 records** |
| --- |
|  |
| 1. exp patient reported outcome measures/ or (((patient* or self) adj1 (report* or rate or rating or rated)) and ((report* or rate or rating or rated) adj3 (outcome* or measur* or symptom*))).ab,ti,kf or ((patientreport* or selfreport*) adj3 (outcome* OR measur* OR symptom*)).ab,ti,kf or (prom or proms or promis).ab,ti,kf |
| 2. exp "treatment adherence and compliance"/ OR exp "costs and cost analysis"/ or feasibility studies/ or program evaluation/ or (acceptability or satisfaction or experience* or adoption or uptake* or appropriateness or cost? or feasibilty or barrier* or challeng* or facilitate* or impact* or implement* or compliance or adherence or fidelity or sustainab* or (success* adj1 factor*) or (lesson* adj1 learned)).ab,ti,kf |
| 3. ((clinical or routine or regular*) and (care or practice or assess* or implement* or utiliz* or measure* or collect*)).ab,ti,kf. |
| 4. exp health status disparities/ or exp healthcare disparities/ or exp health services accessibility/ or (disparit* or inequalit* or equity).ab,ti,kf |
| 5. minority groups/ or minority health/ or ethnic groups/ or hispanic americans/ or african americans/ or indians, north american/ or inuits/ or asian americans/ or (minorities or minority group* or racial or race or ethnic* or latino* or latina* or latinx or hispanic* or mexican american? or latin american? or black? or african american? or non?white or native american* or american indian* or first nation).ab,ti,kf |
| 6. exp social determinants of health/ or socioeconomic factors/ or economic status/ or educational status/ or working poor/ or exp poverty/ or exp social class/ or social factors/ or (disadvantaged or impoverished or poverty or working poor or income).ab,ti,kf or ((social or socio*) adj1 (background or status or position or class)).ab,ti,kf |
| 7. rural population/ or urban population/ or residence characteristics/ or public housing/ or (rural or urban or neighborhood* or geographic).ab,ti,kf |
| 8. exp literacy/ or exp health literacy/ or (literacy or literate or illiterate or second language or foreign language).ab,ti,kf or (english adj3 (profic* or abilit*)).ab,ti,kf |
| 9. exp "sexual and gender minorities"/ or homosexuality/ or bisexuality/ or transsexualism/ or (lgbtq or lgbt or lbg or glbt or glbtq or transgender* or trans-gender or transsexual* or trans-person* or trans-adult* OR trans-individual* or trans-patient* or trans-men or trans-women or trans-participant* or trans-youth* or cisgender or cissexual or gay or gays or homosexual* or non-heterosexual* or lesbian* or gender minorit* or sexual minorit*).ab,ti,kf |
| 10. geriatrics/ or (elderly or elder).ab,ti,kf or ((older or aged) adj2 (adult* or people or patient* or individual* or population*)).ab,ti,kf |
| 11. or/4-10 |
| 12. 1 and 2 and 3 and 11 |

| **Embase (Elsevier, 1974 - )**  **Search Date: September 23, 2021**  **1,974 Records** |
| --- |
|  |
| 1. 'patient reported outcome'/exp OR (((patient* OR self) NEAR/1 (report* OR rate OR rating OR rated)):ab,ti,kw AND ((report* OR rate OR rating OR rated) NEAR/3 (outcome* OR measur* OR symptom*)):ab,ti,kw) OR ((patientreport* OR selfreport*) NEAR/3 (outcome* OR measur* OR symptom*)):ab,ti,kw OR (prom OR proms OR promis):ab,ti,kw |
|  |
| 2. 'patient compliance'/de OR 'cost'/de OR 'feasibility study'/exp OR 'program evaluation'/exp OR (acceptability OR satisfaction OR experience* OR adoption OR uptake* OR appropriateness OR cost OR costs OR feasibilty OR barrier* OR challeng* OR facilitate* OR impact* OR implement* OR compliance OR adherence OR fidelity OR sustainab* OR 'success* factor*' OR 'lesson* learned'):ab,ti,kw |
|  |
| 3. ((clinical OR routine OR regular*) AND (care OR practice OR assess* OR implement* OR utiliz* OR measure* OR collect*)):ab,ti,kw |
|  |
| 4. 'health disparity'/exp OR 'health care disparity'/exp OR 'health care access'/de OR 'unmet medical need'/exp OR (disparit* OR inequalit* OR equity):ab,ti,kw |
|  |
| 5. 'minority group'/exp OR 'minority health'/exp OR 'ethnic group'/de OR 'Hispanic'/exp OR 'African American'/exp OR 'American Indian'/exp OR 'Asian American'/exp OR (minorities OR 'minority group*' OR racial OR race OR ethnic* OR latino* OR latina* OR latinx OR hispanic* OR 'mexican america*' OR 'latin american*' OR black OR blacks OR 'african american*' OR 'non-white' OR 'native american*' OR 'american indian*' OR 'first nation'):ab,ti,kw |
|  |
| 6. 'social determinants of health'/exp OR 'educational status'/exp OR 'economic status'/exp OR 'poverty'/exp OR 'income group'/exp OR 'social status'/exp OR (disadvantaged OR impoverished OR poverty OR 'working poor' OR income):ab,ti,kw OR ((social OR socio*) NEXT/1 (background OR status OR position OR class)):ab,ti,kw |
|  |
| 7. 'rural population'/exp OR 'urban population'/exp OR 'neighborhood'/exp OR (rural OR urban OR neighborhood* OR geographic):ab,ti,kw |
|  |
| 8. 'literacy'/exp OR (literacy OR literate OR illiterate OR 'second language' OR 'foreign language'):ab,ti,kw OR (english NEAR/3 (profic* OR abilit*)):ab,ti,kw |
|  |
| 9. 'sexual and gender minority'/exp OR (lgbtq OR lgbt OR lbg OR glbt OR glbtq OR transgender* OR 'trans-gender' OR transsexual* OR 'trans-person*' OR 'trans-adult*' OR 'trans-individual*' OR 'trans-patient*' OR 'trans-men' OR 'trans-women' OR 'trans-participant*' OR 'trans-youth*' OR cisgender OR cissexual OR gay OR gays OR homosexual* OR 'non-heterosexual*' OR lesbian* OR 'gender minorit*' OR 'sexual minorit*'):ab,ti,kw |
|  |
| 10. (elderly OR elder):ab,ti,kw OR ((older OR aged) NEAR/2 (adult* OR people OR patient* OR individual* OR population*)):ab,ti,kw |
|  |
| 11. #4 OR #5 OR #6 OR #7 OR #8 OR #9 OR #10 |
|  |
| 12. #1 AND #2 AND #3 AND #11 |
|  |
| 13. #12 NOT ('conference abstract'/it OR 'conference review'/it) |

| **Web of Science Core Colleciton (Clarivate)**  **Editions = ESCI, SCI-EXPANDED, SSCI**  **Limited to Science and Social Science Citation Indexes, plus Emerging Sources**  **Search Date: September 24, 2021**  **2,712 Records** |
| --- |
|  |
| 1. TS=(((("patient*" OR "self") NEAR/1 ("report*" OR "rate" OR "rating" OR "rated")) AND (("report*" OR "rate" OR "rating" OR "rated") NEAR/3 ("outcome*" OR "measur*" OR "symptom*"))) OR (("patientreport*" OR "selfreport*") NEAR/3 ("outcome*" OR "measur*" OR "symptom*")) OR "prom" OR "proms" OR "promis") |
|  |
| 2. TS=("acceptability" OR "satisfaction" OR "experience*" OR "adoption" OR "uptake*" OR "appropriateness" OR "cost" OR "costs" OR "feasibilty" OR "barrier*" OR "challeng*" OR "facilitate*" OR "impact*" OR "implement*" OR "compliance" OR "adherence " OR "fidelity" OR "sustainab*" OR "success* factor*" OR "lesson* learned") |
|  |
| 3. TS=(("clinical" OR "routine" OR "regular*") AND ("care" OR "practice" OR "assess*" OR "implement*" OR "utiliz*" OR "measure*" OR "collect*")) |
|  |
| 4. TS=("disparit*" OR "inequalit*" OR "equity") |
|  |
| 5. TS=("minorities" OR "minority group*" OR "racial" OR "race" OR "ethnic*" OR "latino*" OR "latina*" OR "latinx" OR "hispanic*" OR "mexican america*" OR "latin american*" OR "black" OR "blacks" OR "african american*" OR "non-white" OR "native american*" OR "american indian*" OR "first nation") |
|  |
| 6. TS=("disadvantaged" OR "impoverished" OR "poverty" OR "working poor" OR "income" OR (("social" OR "socio*") NEAR/1 ("background" OR "status" OR "position" OR "class"))) |
|  |
| 7. TS=("rural" OR "urban" OR "neighborhood*" OR "geographic") |
|  |
| 8. TS=("literacy" OR "literate" OR "illiterate" OR "second language" OR "foreign language" OR ("english" NEAR/3 ("profic*" OR "abilit*"))) |
|  |
| 9. TS=("lgbtq" OR "lgbt" OR "lbg" OR "glbt" OR "glbtq" OR "transgender*" OR "trans-gender" OR "transsexual*" OR "trans-person*" OR "trans-adult*" OR "trans-individual*" OR "trans-patient*" OR "trans-men" OR "trans-women" OR "trans-participant*" OR "trans-youth*" OR "cisgender" OR "cissexual" OR "gay" OR "gays" OR "homosexual*" OR "non-heterosexual*" OR "lesbian*" OR "gender minorit*" OR "sexual minorit*") |
|  |
| 10. TS=("elderly" OR "elder" OR (("older" OR "aged") NEAR/2 ("adult*" OR "people" OR "patient*" OR "individual*" OR "population*"))) |
|  |
| 11. #4 OR #5 OR #6 OR #7 OR #8 OR #9 OR #10 |
|  |
| 12. #1 AND #2 AND #3 AND #11 |

| **CINAHL (EBSCO)**  **Search Date: September 24, 2021**  **964 Records** |
| --- |
|  |
| 1. (MH ("Patient-Reported Outcomes") OR TI ((((patient* OR self) W1 (report* OR rate OR rating OR rated)) and ((report* OR rate OR rating OR rated) N3 (outcome* OR measur* OR symptom*))) OR ((patientreport* OR selfreport*) N3 (outcome* OR measur* OR symptom*)) OR prom OR proms OR promis) OR AB ((((patient* OR self) W1 (report* OR rate OR rating OR rated)) and ((report* OR rate OR rating OR rated) N3 (outcome* OR measur* OR symptom*))) OR ((patientreport* OR selfreport*) N3 (outcome* OR measur* OR symptom*)) OR prom OR proms OR promis)) |
|  |
| 2. (MH ("Patient Compliance" OR "Costs and Cost Analysis+" OR "Program Evaluation" OR "Program Implementation") OR TI (acceptability OR satisfaction OR experience* OR adoption OR uptake* OR appropriateness OR cost? OR feasibilty OR barrier* OR challeng* OR facilitate* OR impact* OR implement* OR compliance OR adherence OR fidelity OR sustainab* OR (success* W1 factor*) OR (lesson* W1 learned)) OR AB (acceptability OR satisfaction OR experience* OR adoption OR uptake* OR appropriateness OR cost? OR feasibilty OR barrier* OR challeng* OR facilitate* OR impact* OR implement* OR compliance OR adherence OR fidelity OR sustainab* OR (success* W1 factor*) OR (lesson* W1 learned))) |
|  |
| 3. (TI ((clinical OR routine OR regular*) and (care OR practice OR assess* OR implement* OR utiliz* OR measure* OR collect*)) OR AB ((clinical OR routine OR regular*) and (care OR practice OR assess* OR implement* OR utiliz* OR measure* OR collect*))) |
|  |
| 4. (MH ("Healthcare Disparities" OR "Health Status Disparities" OR "Health Services Accessibility") OR TI (disparit* OR inequalit* OR equity) OR AB (disparit* OR inequalit* OR equity)) |
|  |
| 5. (MH ("Minority Groups" OR "Ethnic Groups" OR "Hispanic Americans" OR "Native Americans" OR "Black Persons") OR TI (minorities or "minority group*" or racial or race or ethnic* or latino* or latina* or latinx or hispanic* or "mexican american*" or "latin american*" or black OR blacks or "african american*" or "non-white" or "native american*" or "american indian*" or "first nation*") OR AB (minorities or "minority group*" or racial or race or ethnic* or latino* or latina* or latinx or hispanic* or "mexican american*" or "latin american*" or black OR blacks or "african american*" or "non-white" or "native american*" or "american indian*" or "first nation*")) |
|  |
| 6. (MH ("Social Determinants of Health" OR "Socioeconomic Factors" OR "Economic Status" OR "Poverty" OR "Social Class" OR "Social Factors" OR "Educational Status") OR TI (disadvantaged OR impoverished OR poverty OR "working poor" OR income OR ((social OR socio*) W1 (background OR status OR position OR class))) OR AB (disadvantaged OR impoverished OR poverty OR "working poor" OR income OR ((social OR socio*) W1 (background OR status OR position OR class)))) |
|  |
| 7. (MH ("Rural Population" OR "Urban Population" OR "Residence Characteristics") OR TI (rural or urban or neighborhood* or geographic) OR AB (rural or urban or neighborhood* or geographic)) |
|  |
| 8. (MH ("Literacy" OR "Health Literacy") OR TI (literacy or literate or illiterate or "second language" or "foreign language" or (english N3 (profic* or abilit*))) OR AB (literacy or literate or illiterate or "second language" or "foreign language" or (english N3 (profic* or abilit*)))) |
|  |
| 9. (MH ("Sexual and Gender Minorities" OR "Homosexuality" OR "Bisexuality" OR "Transsexualism") OR TI (lgbtq OR lgbt OR lbg OR glbt OR glbtq OR transgender* OR "trans-gender" OR transsexual* OR "trans-person*" OR "trans-adult*" OR "trans-individual*" OR "trans-patient*" OR "trans-men" OR "trans-women" OR "trans-participant*" OR "trans-youth*" OR cisgender OR cissexual OR gay OR gays OR homosexual* OR "non-heterosexual*" OR lesbian* OR "gender minorit*" OR "sexual minorit*") OR AB (lgbtq OR lgbt OR lbg OR glbt OR glbtq OR transgender* OR "trans-gender" OR transsexual* OR "trans-person*" OR "trans-adult*" OR "trans-individual*" OR "trans-patient*" OR "trans-men" OR "trans-women" OR "trans-participant*" OR "trans-youth*" OR cisgender OR cissexual OR gay OR gays OR homosexual* OR "non-heterosexual*" OR lesbian* OR "gender minorit*" OR "sexual minorit*")) |
|  |
| 10. (MH ("Geriatrics") OR TI (elderly OR elder OR ((older OR aged) NEAR/2 (adult* OR people OR patient* OR individual* OR population*)) OR AB (elderly OR elder OR ((older OR aged) NEAR/2 (adult* OR people OR patient* OR individual* OR population*))) |
|  |
| 11. S4 OR S5 OR S6 OR S7 OR S8 OR S9 OR S10 |
|  |
| 12. S1 AND S2 AND S3 AND S11 |

| **PsycINFO (EBSCO)**  **Search Date: September 27, 2021**  **755 Records** |
| --- |
|  |
| 1. (DE ("Patient Reported Outcome Measures") OR TI ((((patient* OR self) W1 (report* OR rate OR rating OR rated)) and ((report* OR rate OR rating OR rated) N3 (outcome* OR measur* OR symptom*))) OR ((patientreport* OR selfreport*) N3 (outcome* OR measur* OR symptom*)) OR prom OR proms OR promis) OR AB ((((patient* OR self) W1 (report* OR rate OR rating OR rated)) and ((report* OR rate OR rating OR rated) N3 (outcome* OR measur* OR symptom*))) OR ((patientreport* OR selfreport*) N3 (outcome* OR measur* OR symptom*)) OR prom OR proms OR promis)) |
|  |
| 2. (DE ("Treatment Compliance" OR "Costs and Cost Analysis" OR "Health Care Costs" OR "Program Evaluation" OR "Program Development") OR TI (acceptability OR satisfaction OR experience* OR adoption OR uptake* OR appropriateness OR cost? OR feasibilty OR barrier* OR challeng* OR facilitate* OR impact* OR implement* OR compliance OR adherence OR fidelity OR sustainab* OR (success* W1 factor*) OR (lesson* W1 learned)) OR AB (acceptability OR satisfaction OR experience* OR adoption OR uptake* OR appropriateness OR cost? OR feasibilty OR barrier* OR challeng* OR facilitate* OR impact* OR implement* OR compliance OR adherence OR fidelity OR sustainab* OR (success* W1 factor*) OR (lesson* W1 learned))) |
|  |
| 3. (TI ((clinical OR routine OR regular*) and (care OR practice OR assess* OR implement* OR utiliz* OR measure* OR collect*)) OR AB ((clinical OR routine OR regular*) and (care OR practice OR assess* OR implement* OR utiliz* OR measure* OR collect*))) |
|  |
| 4. (DE "Health Disparities" OR DE "Racial Disparities" OR "Equity" OR "Health Care Access" OR "Treatment Barriers") OR TI (disparit* OR inequalit* OR equity) OR AB (disparit* OR inequalit* OR equity)) |
|  |
| 5. (DE ("Minority Groups" OR "Racial and Ethnic Groups" OR "Latinos/Latinas" OR DE "Mexican Americans" OR "Blacks" OR "American Indians" OR "Alaska Natives" OR "Inuit") OR TI (minorities or "minority group*" or racial or race or ethnic* or latino* or latina* or latinx or hispanic* or "mexican american*" or "latin american*" or black OR blacks or "african american*" or "non-white" or "native american*" or "american indian*" or "first nation*") OR AB (minorities or "minority group*" or racial or race or ethnic* or latino* or latina* or latinx or hispanic* or "mexican american*" or "latin american*" or black OR blacks or "african american*" or "non-white" or "native american*" or "american indian*" or "first nation*")) |
|  |
| 6. (DE ("Socioeconomic Factors" OR "Economic Disadvantage" OR "Income Level" OR "Social Class" OR "Disadvantaged" OR "Social Disadvantage" OR "Socioeconomic Status" OR "Social Class" OR "Income Level" OR "Poverty" OR "Poverty Areas" OR "Educational Attainment Level") OR TI (disadvantaged OR impoverished OR poverty OR "working poor" OR income OR ((social OR socio*) W1 (background OR status OR position OR class))) OR AB (disadvantaged OR impoverished OR poverty OR "working poor" OR income OR ((social OR socio*) W1 (background OR status OR position OR class)))) |
|  |
| 7. (DE ("Rural Environments" OR "Urban Environments" OR "Neighborhoods" OR "Geography") OR TI (rural or urban or neighborhood* or geographic) OR AB (rural or urban or neighborhood* or geographic)) |
|  |
| 8. (DE ("Literacy" OR "Health Literacy" OR "Language Proficiency") OR TI (literacy or literate or illiterate or "second language" or "foreign language" or (english N3 (profic* or abilit*))) OR AB (literacy or literate or illiterate or "second language" or "foreign language" or (english N3 (profic* or abilit*)))) |
|  |
| 9. (DE ("Sexual Minority Groups" OR "LGBTQ" OR "Homosexuality" OR "Lesbianism" OR "Male Homosexuality" OR "Bisexuality" OR "Transsexualism" OR "Gender Nonconforming" OR "Transsexualism" OR "Transgender") OR TI (lgbtq OR lgbt OR lbg OR glbt OR glbtq OR transgender* OR "trans-gender" OR transsexual* OR "trans-person*" OR "trans-adult*" OR "trans-individual*" OR "trans-patient*" OR "trans-men" OR "trans-women" OR "trans-participant*" OR "trans-youth*" OR cisgender OR cissexual OR gay OR gays OR homosexual* OR "non-heterosexual*" OR lesbian* OR "gender minorit*" OR "sexual minorit*") OR AB (lgbtq OR lgbt OR lbg OR glbt OR glbtq OR transgender* OR "trans-gender" OR transsexual* OR "trans-person*" OR "trans-adult*" OR "trans-individual*" OR "trans-patient*" OR "trans-men" OR "trans-women" OR "trans-participant*" OR "trans-youth*" OR cisgender OR cissexual OR gay OR gays OR homosexual* OR "non-heterosexual*" OR lesbian* OR "gender minorit*" OR "sexual minorit*")) |
|  |
| 10. (DE ("Geriatrics") OR TI (elderly OR elder OR ((older OR aged) NEAR/2 (adult* OR people OR patient* OR individual* OR population*)) OR AB (elderly OR elder OR ((older OR aged) NEAR/2 (adult* OR people OR patient* OR individual* OR population*))) |
|  |
| 11. S4 OR S5 OR S6 OR S7 OR S8 OR S9 OR S10 |
|  |
| 12. S1 AND S2 AND S3 AND S11 |
