# Supplementary material for: Implementing patient-reported outcomes in routine clinical care for diverse and underrepresented patients in the United States
Source: J Patient Rep Outcomes. 2022 Mar 7;6:20. doi: 10.1186/s41687-022-00428-z (PMC8901833; doi:10.1186/s41687-022-00428-z)
Supplement: Supplementary file 2 — Additional file 2: Table S2. Details of the specific implementation outcomes by studies included in the review. [file 41687_2022_428_MOESM2_ESM.docx]

**Additional file 2: Table S2.** Details of the specific implementation outcomes by studies included in the review.

| **Study authors** | **Acceptability** | **Adoption** | **Appropriateness** | **Costs** | **Feasibility** | **Fidelity** | **Penetration** | **Sustainability** |
| --- | --- | --- | --- | --- | --- | --- | --- | --- |
| Anderson, J et al.^36^ | Identified body map, free-text option, patient dashboard, social support resources, electronic pillbox, and loss-framed incentive plan to be used in intervention; identified changes to language (to be patient-friendly) and app aesthetics |  | Overall, focus group participants expressed positive views of the app and its features, particularly the potential to improve patient-provider communication/AET adherence   Nurses reported the app increased the potential for patient-provider communication and improved ability to quickly address patient's concerns |  | Nurses reported that app did not interrupt clinical workflow or increase work burden |  |  |  |
| Anderson, K et al.^37^ |  | 82% of eligible patients enrolled  84% of intervention and 72% of control completed first follow-up assessment |  |  | 68% of intervention and 86% of control completed second follow-up assessment   71% of IVR assessments completed successfully  33% of physicians reported symptom feedback influenced clinical decisions |  |  |  |
| Arcia et al.^26^ | All 10 Phase II participants demonstrated complete gist and verbatim comprehension for both infographics   All patients wanted infographic every time they had an ACQ score or PFT; a few patients indicated they would also want the information verbally |  | Participants reacted very favorably to infographics and expressed intentions to engage in self-management behaviors |  | All 10 Phase II participants demonstrated complete gist and verbatim comprehension for both infographics |  |  |  |
| Calamia et al.^46^ |  |  |  |  | A minority of participants required assistance with the computer and/or providing further clarifications to the questions and tasks | High correlation between web-based measures and paper and pencil measure; high re-rest correlation after 2 weeks |  |  |
| Gabbard et al.^49^ | Majority of older adults felt the use of the K-Pal application was easy to use; some participants were overwhelmed with the amount of questionnaires, especially during initial use; suggestions for numbering the questionnaires and the addition of an intro tutorial video | 4.5% withdrew after baseline assessment, no longer interested in participation |  |  | Due to patient fatigue from HD treatment, some patients were rescheduled to complete the K-Pal app during upcoming treatments  9% withdrew after 3-month follow-up assessment, no longer interested in participation |  |  |  |
| Gonzalez et al. (1995)^33^ | Preference for computer-telephone method vs. face-to-face:  60% of Spanish speakers vs. 76% of English speakers |  |  |  | Telephone administration time significantly longer than the face-to-face method for both samples |  |  |  |
| Gonzalez et al. (2000)^34^ | The majority (90%) of both samples preferred the face-to-face method compared to the computerized method |  |  |  |  |  |  |  |
| Hahn et al.^38^ |  | 26.6% refused to participate |  | Software: $2000/year. Cost per computer: $2100; Further study is required, to determine cost-effectiveness | Nearly 100% completion rate; average completion time was 33 and 28 min for low and high literacy patients, respectively, and over 80% in each group said that the assessment was not too long  31% did not require any help using the touchscreen, including seven who had never used a computer before; 59% required some assistance, and 10% required “a lot” of assistance |  |  |  |
| Hinami et al.^28^ |  | 10% refused to participate |  |  | 14% failed to start the survey and another 29 % omitted key items. Patients who were unable to complete at least one of these instruments were more likely to be non-Hispanic Black 34 vs. 28%, respectively, and older 64 vs. 57, respectively. |  |  |  |
| Hirsh et al.^45^ | 52% found the MDHAQ-PGA-VAS confusing  42% found the DAS28-PGA-VAS confusing  Significantly more found the wording harder to understand and were more unclear if the question was regarding RA or general health for the MDHAQ-PGA-VAS  Depression was a predictor for confusion |  |  |  | 50% of subjects did not check a single box on questionnaire  Spanish speakers more likely to want help completing the MDHAQ-PGA-VAS |  |  |  |
| Jacoby et al.^50^ | 72% found the survey to be convenient;   44% said they liked that they could "do it on their own time"; 44%  found it easier to talk about things that are difficult;   17% said it’s one way to communicate but they would have preferred speaking to someone in person     83% of respondents found the survey easy; 11%  somewhat easy   ; 6% neither easy nor difficult;   0% somewhat difficult; and 0% difficult. | Most study participants were willing to complete a survey similar to the one conducted in the study when discharged from the hospital. With 14 (77%) saying Yes, 3 (17%) saying No, and 1 (6%) saying  I’m not sure | 0% found no benefit |  | 94% said they had enough time to complete the survey.    Response rates for 12 automated surveys ranged from 84–92%. |  |  |  |
| Jiwani et al.^29^ |  | 100% of all of the 26 patients completed the baseline PROMIS tool |  |  | 100% of all of the 26 patients completed 3- and 6-month assessments on PROMIS tools. Retention rate of 92% after 6 months. |  |  |  |
| Kasturi et al.^42^ | 75.9% liked aspects of the survey  27.9% reported a challenge or suggested improvement (content of questions, technical issues, symptoms during survey completion) | 3.8% declined participation (44% time constraints; 22% discomfort with computers; 11% discomfort with research, 11% did not want to think about disease; 11% injured) | Qualitative responses regarding 'fit' of questions |  | 80.4% completed surveys remotely  Time constraints cited as most frequent reason for not completing surveys  32.8% required reminders (of these, 61.2% required one, 25.4% required two, 13.4% required three)  73 PROMIS items took 11.3 mins to complete |  |  |  |
| Lapin et al.^27^ | Factors associated with a more favorable PROM experience: male sex, nonwhite, younger age, lower income, depression |  | Nonwhite, low income, female, and depressed patients found PROM questions useful |  | Nonwhite patients had increasing satisfaction with care when believing PROMs improved communication and control of care |  |  |  |
| Liu et al.^43^ | Most patients wanted to see their own data expressed in a dashboard at a future appointment  Most patients preferred the dashboard with more detailed information and ability to customize the dashboard  Interest in expanding use to include resources and communication systems | Some clinicians expressed significant skepticism that they would use the dashboard during their visits | Spanish-speaking and limited health literacy participants more likely to consider the information new or more accessible |  | Limited health literacy patients more likely to find dashboard difficult to understand  Limited health literacy and Spanish-language groups had more difficulty understanding longitudinal nature of data   Spanish-speaking patients did not anticipate challenges in using the dashboard with an interpreter | Clinicians worried about accuracy of EHR data imported into the dashboard |  |  |
| Loo et al.^30^ | Patients generally satisfied (focus group of 8)  Providers also satisfied and 67% indicated that ePROs reduced paperwork burden  Patients appreciated answering challenging questions in a non-stigmatized way | Determined completion rates during initial implementation as well as over time (feasibility) |  | Minimal costs needed after initial implementation phase (program manager shifted to part-time) | ePRO proved time-saving within existing clinic workflows  Over one year, completion of at least one ePRO increased from 66% to 74% |  | Number or providers interfacing with ePRO system increased over time (3 providers to 25 providers) | ePRO program able to be maintained over several years beyond initial implementation |
| Munoz et al.^35^ | The majority of Spanish-speaking (63.2%) preferred computerized version  Reasons for computerized preference: easier and more understandable (42%); interesting, novel, entertaining (25%)  The majority of English-speaking (73.7%) preferred computerized version |  |  |  | Mean time for written survey: 5.3 mins (Spanish) vs. 3.5 mins (English)  Mean time for computerized survey: 12.1 mins (Spanish vs. 10.4 mins (English) | 95% Spanish-speaking responses correctly recognized and scored vs. 99% English-speaking responses |  |  |
| Nyirenda et al.^51^ | Preference to use finger over stylus for input  Some frustrations over glare of the tablet, security pop-ups, and aesthetic design  A majority found the app easy to use and convenient | 84% completed the initial assessment  A majority would use assessment again | A majority of patients founds the app useful |  | 9.5% completed the follow-up assessment   Mean assessment completion time: 8.3 minutes (no difference with race, ethnicity, gender, education); older age associated with longer completion time   A majority of clinicians found it integrated well |  |  |  |
| Ramsey et al.^47^ | Patient satisfaction increased over time  Patients were moderately comfortable  Majority (60% at time 1 and 69% at time 2) were fairly or very satisfied  Majority (71% at time 1 and 73% at time 2) were fairly or very comfortable | EMA response rate was 46% at time 1 | 18% self-reported perfect adherence at time 1 vs. 16% at time 2 |  | 76% completed one or more surveys   EMA response rate was 48% at time 2  All participants missed at least 1 assessment  Non-adherence: busy/bad timing; not having phone when needed; not hearing alarm; technical difficulties; user error |  |  |  |
| Samuel et al.^39^ | Black participants less likely to report ease of use of web-based format (71.4% Black vs. 91.7% White)  Nearly all Black and White respondents were very satisfied/satisfied with the web-based format  Some Black participants preferred paper-and-pen option | Black patients more likely to select the automated telephone system (40% vs. 10.2% White) but most selected web-based (60% vs. 85.7%) overall  Majority would use the app again if returning to clinic (88.9% Black vs. 88% White) | Majority of participants found it very helpful/helpful in reminding them of symptoms (77.8% Black vs. 76% White)  Black patients more likely to report how the ePRO facilitated communication |  | Most reported that their doctors communicated with them about ePRO survey results (88.9% Black vs. 84% White)  Black patients were less likely to understand all survey questions (55.6% Black vs. 96% White) and the summary report (37.5% Black vs. 72% White) |  |  |  |
| Sarkar et al.^32^ | Qualitatively reported frustration, lack of confidence, but also interest in technology to support self-management |  |  |  | 51% able to complete data entry across all apps without assistance  43% completed data retrieval tasks across 11 apps |  |  |  |
| Scholle et al.^11^ | Most patients (unspecified) found PROMs to be acceptable  Some challenges noted to understanding items | 26.2% of the target population participated  Older patients aged 65 or older were less likely to participate in initial PROM collection   Minority patients less likely to participate | Some patients (unspecified) had concerns about relevance  Patients (unspecified) viewed PROMs as a way to increase engagement |  | Older patients aged 65 years and older less likely to complete all PROMIS items  Minority participants less likely to provide complete responses   Patients with Spanish language preference less likely to provide complete responses (OR 0.36)  Variable views from care members between the sites regarding feasibility (one site saw redundancy) |  |  |  |
| Shipp et al.^52^ |  | Factors associated with late response: non-White, non-Asian, older than 81, unemployed/retired/disabled, income less than $70,000, education of high school or less, no transportation access  Late responders more likely to be male, non-White, older than 40, income less than $70,000, education of high school or less |  |  | 69.7% intake forms completed at least 1 hour before the appointment time; 1.2% completed after |  |  |  |
| Smith et al.^40^ |  | 100% completed at least 1 PRO measure and 99% completed all 7 PRO measures at the first timepoint  Overall, most selected web-based versus telephone-based formats, but 38% of Black patients vs. 13% of White patients selected telephone-based |  |  | 67% completed at least 1 PRO measure at all time points, with Black patients less likely (55% vs. 74% White)  63% completed all 7 PRO measures at each time point, with Black patients less likely (52% vs. 70% White)   Black patients less likely to complete surveys at follow-up |  |  |  |
| Stonbraker et al.^31^ | 68.4% preferred the bar over line graph and 56.2% found bar graphs easier to understand (phase 1)  94.3% preferred reports using emojis |  |  |  | Greatest proportion of correct interpretation occurred for the scatterplot visualization  Most willing to share their reports with their providers  Bar graph with emojis at the highest level of comprehension | Some confusion noted qualitatively: a "sad" emoji face was equated with a depression question as opposed to neuropathy because of poor understanding of what neuropathy meant |  |  |
| Wahl et al.^44^ |  | 97% had PROMIS scores recorded |  |  | PROMIS scores recorded at 89% of encounters  Found implementation feasible, even among non-English speakers |  |  |  |
| Wolford et al.^48^ | Computer-only sample: 96% "liked it"  In-person-only sample: 95% "liked it"  Mixed sample: 43% prefer computer, 37% prefer in-person, 20% no preference  Younger patients preferred the computer | 8% of participants declined to participate (6 unwilling to use computer, 9 unwilling to see an interviewer, 12 unwilling to give blood) |  | Computer-assisted interview: $47 per interview  In-person interview: $90 per interview | No significant difference in time to completion; data was processed faster for CAI  Within interview, 100% completed surveys for both computer and in-person interviews  5% dropped out before first and second interview sessions (1 disliked computer, 2 disliked interviewer) |  |  |  |
| Zullig et al.^41^ |  | 77% response rate |  |  | Approximately 2-3 minutes needed for completion |  |  |  |
